# Exploring the nonlinear association between serum uric acid and the TG/HDL-C Ratio: insights from school-aged children with adenoid or tonsillar hypertrophy

**DOI:** 10.3389/fpubh.2026.1613538

**Published:** 2026-01-21

**Authors:** Liming Liu, Yiqing Yang, Ying Liu, Yingyi Chen, Ziyin Zhang, Zhimin Wang, Xin Liu

**Affiliations:** 1 Department of Cardiology, The First Affiliated Hospital of Zhengzhou University, Zhengzhou University, Zhengzhou, Henan, China; 2 Department of Clinical Laboratory, College of Laboratory Medicine, Hebei North University, Zhangjiakou, Hebei, China; 3 Department of Medical Laboratory, The Affiliated Cancer Hospital of Zhengzhou University, Zhengzhou, Henan, China; 4 Central Laboratory, The Affiliated Cancer Hospital of Zhengzhou University & Henan Cancer Hospital, Zhengzhou, Henan, China; 5 Department of Information, The First Affiliated Hospital of Zhengzhou University, Zhengzhou, Henan, China; 6 Department of Endocrinology and Metabolic Diseases, The First Affiliated Hospital of Zhengzhou University, Zhengzhou, Henan, China; 7 Department of Clinical Laboratory, Key Clinical Laboratory of Henan Province, The First Affiliated Hospital of Zhengzhou University, Zhengzhou, Henan, China

**Keywords:** metabolic dysfunction, non-linear relationship, school-age children, serum uric acid, TG/HDL-C ratio

## Abstract

**Background:**

Serum uric acid (SUA) plays a critical role in metabolic dysfunction, including insulin resistance (IR) and lipid dysregulation. This study investigates the association between SUA and the triglyceride-to-high-density lipoprotein cholesterol (TG/HDL-C) ratio in school-aged children with adenoid or tonsillar hypertrophy.

**Methods:**

A single-center retrospective study of 3,026 school-aged children with adenoid or tonsillar hypertrophy was conducted. Baseline characteristics, metabolic markers, and the TG/HDL-C ratio were analyzed across SUA quartiles. Spearman’s test was used to explore the associations between clinical parameters and the TG/HDL-C ratio. A restricted cubic splines (RCS) linear regression model was used to explore the nonlinear relationships between SUA and the TG/HDL-C ratio.

**Results:**

SUA levels were positively associated with the TG/HDL-C ratio (*r* = 0.232, *p* < 0.001). RCS analysis revealed a significant nonlinear relationship between SUA and the TG/HDL-C ratio (*p* for nonlinear = 0.019). When SUA exceeded 4.40 mg/dL, the positive relationship between SUA and the TG/HDL-C ratio became stronger compared to levels below this threshold. Moreover, children with SUA levels >4.40 mg/dL showed a significantly higher proportion of BMI *z*-scores >2.0 and a greater prevalence of dyslipidemia than those with lower SUA levels. The relationship between SUA and the TG/HDL-C ratio in males and females was consistent with that observed in the general population.

**Conclusion:**

Our study identified a nonlinear relationship between SUA levels and the TG/HDL-C ratio in school-age children with adenoid or tonsillar hypertrophy. This nonlinear pattern persisted in sex-stratified analyses, with males exhibiting a higher SUA inflection point compared to females. These findings suggest that SUA could serve as a practical marker for early metabolic risk assessment.

## 1 Introduction

Uric acid is the final product of purine metabolism and has been extensively studied for its pivotal role in metabolic syndrome (1), insulin resistance (IR) (2), type 2 diabetes (3), and cardiovascular disease (4). Recently, the relationship between SUA and the TG/HDL-C ratio has garnered growing research interest (5). Investigating these relationships is crucial, as SUA and the TG/HDL-C ratio collectively reflect interconnected but distinct aspects of metabolic health, offering deeper insights into the mechanisms of metabolic dysfunction.

The TG/HDL-C ratio reflects the balance between triglycerides and protective HDL cholesterol, offering insights into lipid dysregulation and inflammatory states. It has been demonstrated to be strongly correlated with the homeostasis model assessment of IR (5) and is increasingly used to predict metabolic disorders, such as type 2 diabetes mellitus (6) and cardiovascular diseases (7). Understanding how SUA levels correlate with the TG/HDL-C ratio could clarify the bidirectional relationship between uric acid metabolism, insulin resistance, and lipid abnormalities, which is critical for early detection and intervention.

Children with adenoid or tonsillar hypertrophy represent a distinctive pediatric population characterized not only by upper airway obstruction and intermittent hypoxia but also by chronic low-grade inflammation that may predispose them to metabolic dysregulation (8). Increasing evidence suggests that obstructive sleep-related breathing disorders can disrupt lipid homeostasis and impair high-density lipoprotein (HDL) function through systemic inflammation and oxidative stress. Clinically, children with adenoid or tonsillar hypertrophy have significantly lower HDL-C levels, which correlate inversely with adenoidal or tonsillar size and the apnea-hypopnea index, indicating a link between airway obstruction and lipid imbalance (9). Hypoxia-induced metabolic alterations promote the accumulation of purine intermediates and ultimately increase uric acid production. Collectively, these mechanisms suggest that this population is particularly susceptible to disturbances in both lipid metabolism and uric acid regulation.

Overall, few studies have explored the relationship between SUA and the TG/HDL-C ratio in children with adenoid or tonsillar hypertrophy who are at increased metabolic risk. Therefore, our study aimed to elucidate the nonlinear association between these two key metabolic indicators to provide new insights into early identification and intervention for metabolic abnormalities in this special pediatric population.

## 2 Method

### 2.1 Study population

**Figure 1:**
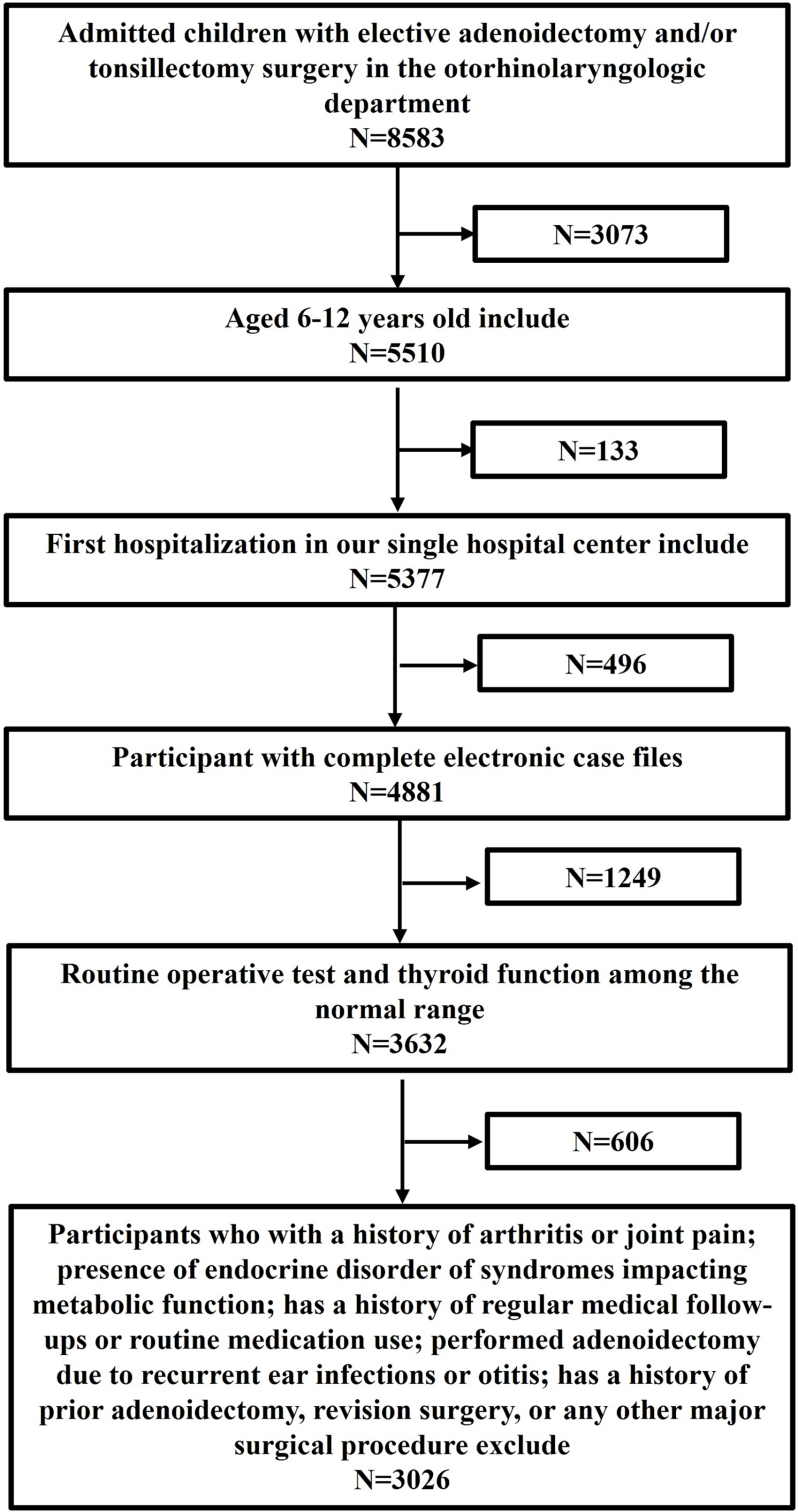
Flowchart of the inclusion and exclusion criteria.

We conducted a retrospective review of the electronic medical records of children aged 6–12 years who underwent elective adenoidectomy and/or tonsillectomy at the Otorhinolaryngology Department of the First Affiliated Hospital of Zhengzhou University. We extracted data from the first page of medical records spanning January 2018 to August 2022, identifying cases using the International Classification of Diseases (ICD-10), along with the Current Procedural Terminology and Healthcare Common Procedure Coding System codes. A total of 8,583 patients were admitted during this period, of whom 3,026 (1,938 boys and 1,088 girls) met the inclusion and exclusion criteria (Figure 1). The study was conducted in accordance with the Declaration of Helsinki, approved by the Ethics Committee of the First Affiliated Hospital of Zhengzhou University (Approval No: 2022-KY-1335). As this research was based on a retrospective review of existing medical records and involved no direct patient intervention, and as all data were fully anonymized before analysis, the requirement for individual informed consent was waived by the Ethics Committee.

### 2.2 Inclusion criteria

The inclusion criteria for the study were as follows: (1) age between 6 and 12 years; (2) first-time hospitalization at the First Affiliated Hospital of Zhengzhou University; (3) availability of routine preoperative test results, including complete blood count, urinalysis, electrolyte levels, liver enzymes [alanine aminotransferase (ALT), aspartate aminotransferase (AST), alkaline phosphatase (ALP), gamma-glutamyl transferase (GGT)], and kidney function markers [serum creatinine (Cr) and blood urea nitrogen (BUN)], all within normal ranges adjusted for sex and age; (4) thyroid function markers (free triiodothyronine, free thyroxine, and thyroid-stimulating hormone) within normal ranges for sex and age; and (5) adenoidectomy conducted due to adenoid and tonsillar hypertrophy.

### 2.3 Exclusion criteria

The exclusion criteria for the study were as follows: (1) incomplete electronic records, especially missing laboratory data; (2) a history of arthritis or joint pain, including gout; (3) presence of endocrine disorders or syndromes impacting metabolic function, such as conditions of the endocrine pancreas, pituitary, adrenal glands, thyroid, or parathyroid gland; (4) a history of regular medical follow-ups or routine medication use; (5) adenoidectomy performed due to recurrent ear infections or otitis; and (6) a history of prior adenoidectomy, revision surgery, or any other major surgical procedure.

### 2.4 Data collection and laboratory measurement

Demographic information, including age, sex, weight, and height, was collected from all participants. The body mass index (BMI) *z*-score was calculated based on the World Health Organization (WHO) growth standards for children aged 2–5 years and the WHO growth reference for those aged 5–19 years using the Age-based Pediatric Growth Reference Charts website (10).

All blood samples were obtained after overnight fasting as part of routine preoperative laboratory testing. Biochemical parameters included serum uric acid (SUA), fasting plasma glucose (FPG), total cholesterol (TC), triglycerides (TG), high-density lipoprotein cholesterol (HDL-C), low-density lipoprotein cholesterol (LDL-C), alanine aminotransferase (ALT), aspartate aminotransferase (AST), gamma-glutamyl transferase (GGT), alkaline phosphatase (ALP), BUN, and creatinine (Cr). These measurements were performed using a standardized automatic biochemistry analyzer (Roche, Germany). Hematological parameters were obtained from a complete blood count (CBC), including white blood cell (WBC), neutrophil, lymphocyte, and monocyte counts. These hematological indices were further used to calculate the derived inflammatory markers. CBC testing was performed using an automated hematology analyzer (Mindray, China) following standardized operational protocols. Internal quality control procedures were applied throughout the testing process, and the coefficient of variation remained below 5%. The following CBC-derived inflammatory indicators were calculated: NLR, dNLR, MLR, NMLR, SIRI, and SII, using the following formulas: NLR = neutrophil counts/lymphocyte counts, dNLR = neutrophil counts/(white blood cell counts − lymphocyte counts), MLR = monocyte counts/lymphocyte counts, NMLR = (monocyte counts + neutrophil counts)/lymphocyte counts, SIRI = neutrophil counts × monocyte counts/lymphocyte counts, and SII = platelet counts × neutrophil counts/lymphocyte counts. The TG/HDL-C ratio was calculated as TG (mmol/L) divided by HDL-C (mmol/L) (11).

### 2.5 Serum uric acid quartile grouping

We categorized all participants into quartiles based on their serum uric acid (SUA) levels: Q1 (SUA ≤ 3.66 mg/dL), Q2 (3.66 mg/dL < SUA ≤ 4.28 mg/dL), Q3 (4.28 mg/dL < SUA ≤ 5.02 mg/dL), and Q4 (SUA > 5.02 mg/dL).

### 2.6 The definition of dyslipidemia

Participants were defined to have dyslipidemia if they met any of the following conditions: TC ≥ 5.20 mmol/L; TG ≥ 1.10 mmol/L for children younger than 10 years or ≥1.50 mmol/L for those aged 10 years or older; HDL-C < 1.00 mmol/L; LDL-C ≥ 3.40 mmol/L; or non-HDL-C ≥ 3.70 mmol/L (12).

### 2.7 Statistical analysis

Continuous variables with a normal distribution are expressed as mean ± standard deviation (mean ± SD), whereas non-normally distributed continuous variables are presented as median (P25, P75). Categorical variables are summarized as percentages. For multiple comparisons of normally distributed variables, pairwise between-group analyses were conducted using the Bonferroni correction when variances were homogeneous and the Dunnett T3 method when variances were unequal. For multiple comparisons of non-normally distributed variables, the Kruskal–Wallis H test was employed, followed by *post-hoc* pairwise comparisons using the Dunn–Bonferroni correction. Spearman’s test was used to calculate the correlation coefficient (rho) between the TG/HDL-C and clinical parameters. Correlation values of *p* value of <0.05 were considered statistically significant. Restricted cubic spline (RCS) regression models were used to examine the nonlinear relationship between SUA and the TG/HDL-C ratio. The RCS model incorporated 4 knots placed at the 5th, 35th, 65th, and 95th percentiles of the variable distribution. All RCS analyses were adjusted for potential confounders, including age, BMI *z*-score, sex, ALT, AST, GGT, and ALP levels. To further quantify the inflection point suggested by the RCS models, we fitted two piecewise linear regression models using data-driven grid search. For each candidate SUA value (increment of 0.1 mg/dL), we constructed a two-segment linear model and selected the inflection point that minimized the Akaike Information Criterion (AIC). Non-parametric bootstrap resampling (500 iterations) was used to estimate the sampling distribution of the inflection point and to derive the 95% confidence intervals. All statistical tests were two-sided, and a *p* value of <0.05 was considered statistically significant. Statistical analyses were performed using SPSS version 21.0 for Windows (SPSS Inc., Chicago, IL) and R version 4.4.1.^1^

## 3 Results

### 3.1 Characteristics of the study population

The baseline characteristics of the study participants are summarized in Table 1. A total of 3,026 participants were included, with approximately 64.0% being male. The mean age of the cohort was 8.4 ± 1.9 years, and the overall mean SUA level was 4.41 ± 1.06 mg/dL. Mean SUA levels for each quartile were as follows: Quartile 1, 3.20 ± 0.36 mg/dL; Quartile 2, 3.98 ± 0.17 mg/dL; Quartile 3, 4.62 ± 0.21 mg/dL; and Quartile 4, 5.83 ± 0.77 mg/dL. Participants’ age increased progressively across SUA quartiles (*p* < 0.001). There was no significant difference in the male-to-female ratio between the quartile groups (*p* = 0.060). The mean TG/HDL-C ratio was 1.64 ± 1.38, with values of 1.37 ± 1.12, 1.48 ± 1.15, 1.65 ± 1.38, and 2.07 ± 1.6 across the quartiles (*p* < 0.001). As the SUA quartile increased, significant upward trends were observed for several metabolic markers, including BMI *z*-score, Cr, BUN, TC, TG, LDL-C, and ALT (*p* < 0.050 for all). Conversely, HDL-C levels showed a significant decrease with increasing SUA levels (*p* < 0.001). Notably, FPG levels showed no significant variation across quartiles (*p* = 0.096).

**Table 1:** Characteristics of the study population according to the SUA quartiles.

	The SUA level					
Characteristics	Total	Quartile 1 <3.67	Quartile 2 3.67 ~ 4.28	Quartile 3 4.28 ~ 5.02	Quartile 4 5.02≥	*p-*value
*N*	3,026	758	753	758	757	–
Boys [*n*(%)]	1938 (64.0)	472 (62.3)	471 (62.5)	480 (63.3)	515 (68.0)	0.060
Age, years	8.4 ± 1.9	7.8 ± 2.5	8.0 ± 1.7	8.4 ± 1.8	9.2 ± 2.1	<0.001
BMI *z*-score	0.38 ± 1.10	0.11 ± 1.14	0.26 ± 1.12	0.44 ± 1.05	0.73 ± 0.98	<0.001
SUA, mg/dL	4.41 ± 1.06	3.20 ± 0.36	3.98 ± 0.17	4.26 ± 0.21	5.38 ± 0.77	<0.001
FPG, mmol/L	4.50 (4.23, 4.78)	4.48 (4.22, 4.71)	4.21 (4.51,4.79)	4.51 (4.25,4.80)	4.52 (4.23, 4.81)	0.096
Cr, μmol/L	41.00 (36.00, 46.00)	38.50 (34.00, 43.00)	41.00 (36.00, 45.00)	42.00 (37.00, 47.00)	43.00 (39.00, 49.00)	<0.001
BUN, mmol/L	4.60 (3.90, 5.37)	4.50 (3.90, 5.27)	4.50 (3.82, 5.39)	4.62 (4.00, 5.40)	4.70 (4.00, 5.40)	0.008
TC, mmol/L	3.77 (3.37, 4.22)	3.69 (3.30, 4.15)	3.81 (3.41, 4.26)	3.80 (3.39, 4.23)	3.79 (3.34, 4.23)	0.001
TG, mmol/L	0.77 (0.59, 1.06)	0.71 (0.55, 0.95)	0.75 (0.57, 1.01)	0.76 (0.60, 1.05)	0.90 (0.67, 1.30)	<0.001
HDL-C, mmol/L	1.39 (1.20, 1.60)	1.45 (1.25, 1.66)	1.43 (1.24, 1.63)	1.38 (1.21, 1.56)	1.31 (1.12, 1.49)	<0.001
LDL-C, mmol/L	2.07 (1.74,2.44)	1.99 (1.66, 2.31)	2.07 (1.78, 2.44)	2.14 (1.76, 2.49)	2.09 (1.76, 2.48)	<0.001
AST, U/L	19.00 (22.00, 24.00)	22.00 (19.00, 25.00)	22.00 (19.00, 25.00)	22.00 (19.00, 24.00)	20.00 (18.00, 23.00)	<0.001
ALT, U/L	12.00 (10.00, 16.00)	12.00 (10.00, 14.00)	12.00 (10.00, 15.00)	12.50 (10.00, 16.00)	14.00 (11.00, 17.00)	<0.001
ALP, U/L	244.00 (209.00, 286.00)	234.50 (200.00, 271.00)	237.00 (204.00, 274.00)	245.00 (212.00, 290.00)	261.00 (223.00, 313.00)	<0.001
GGT, U/L	11.00 (9.00, 13.00)	10.00 (8.00, 11.00)	10.00 (9.00, 12.00)	11.00 (9.00, 13.00)	12.00 (10.00, 15.00)	<0.001
White blood cells (10^3^/μL)	6.70 (5.75, 7.85)	6.55 (5.63, 7.62)	6.70 (5.70, 7.89)	6.65 (5.76, 7.80)	6.90 (5.93, 8.00)	<0.001
Neutrophils (10^3^/μL)	3.01 (2.40, 3.86)	2.85 (2.28, 3.67)	2.92 (2.33, 3.69)	2.97 (2.44, 3.79)	3.25 (2.60, 4.12)	<0.001
Monocyte (10^3^/μL)	0.47 (0.38, 0.58)	0.46 (0.38, 0.58)	0.47 (0.38, 0.58)	0.46 (0.38, 0.57)	0.48 (0.40, 0.60)	0.019
Lymphocyte (10^3^/μL)	2.79 (2.29, 3.38)	2.80 (2.30, 3.35)	2.79 (2.31, 3.50)	2.80 (2.34, 3.35)	2.77 (2.23, 3.31)	0.124
Platelet (10^3^/μL)	293.00 (254.00, 336.00)	286.00 (249.00, 329.00)	294.00 (253.00, 340.00)	296.00 (256.00, 338.00)	298.00 (256.00, 341.00)	0.003
NLR	1.06 (0.81, 1.44)	1.00 (0.76, 1.37)	1.03 (0.77, 1.38)	1.06 (0.83, 1.40)	1.17 (0.91, 1.59)	<0.001
dNLR	0.811 (0.77, 0.84)	0.81 (0.77, 0.84)	0.81 (0.77, 0.84)	0.81 (0.78, 0.84)	0.82 (0.78, 0.85)	<0.001
MLR	0.17 (0.13, 0.21)	0.17(0.13, 0.21)	0.16 (0.13, 0.21)	0.13 (0.16, 0.21)	0.17 (0.13, 0.23)	0.001
NMLR	1.24 (0.96, 1.64)	1.17(0.90, 1.57)	1.89 (0.90, 1.60)	1.23(0.98, 1.59)	1.37 (1.06, 1.80)	<0.001
SIRI (10^3^/μL)	0.50 (0.34, 0.74)	0.45 (0.32, 0.71)	0.47 (0.33, 0.69)	0.50 (0.35, 0.72)	0.56 (0.39, 0.83)	<0.001
SII (10^3^/μL)	312.15 (226.86, 442.39)	283.07 (208.9, 408.36)	305.26 (214.46, 420.69)	315.33 (237.87, 439.26)	347.03 (255.88, 488.63)	<0.001
TG/HDL-C ratio	1.64 ± 1.38	1.37 ± 1.12	1.48 ± 1.15	1.65 ± 1.38	2.07 ± 1.68	<0.001

As shown in Table 1, increasing SUA quartiles from Q1 to Q4 were accompanied by a clear upward trend in multiple hematological parameters. Participants in the higher SUA quartiles showed progressively higher WBC, neutrophil, monocyte, and platelet counts. Consistently, all CBC-derived inflammatory indices, including NLR, dNLR, MLR, NMLR, SIRI, and SII, demonstrated a stepwise increase across SUA quartiles, suggesting a gradual enhancement of the systemic inflammatory status associated with elevated SUA levels.

### 3.2 The relationship between TG/HDL-C and clinical parameters

Table 2 presents the correlations between clinical parameters and the TG/HDL-C ratio. In the overall population, TG/HDL-C showed significant positive correlations with age (*r* = 0.201, *p*<0.001), BMI *z*-score (*r* = 0.168, *p* < 0.001), SUA (*r* = 0.232, *p* < 0.001), LDL-C (*r* = 0.152, *p* < 0.001), and liver enzyme levels, including ALT (*r* = 0.171, *p* < 0.001), ALP (*r* = 0.138,) and GGT (*r* = 0.277, *p* < 0.001). Conversely, AST exhibited a modest negative correlation with the TG/HDL-C ratio (*r* = −0.176, *p* < 0.001).

**Table 2:** Clinical parameters associated with the TG/HDL-C ratio in the subjects.

Variables	Total		Male		Female	
	*r*	*p-*value	*r*	*p*-value	*r*	*p-*value
Age (years)	0.201	<0.001	0.213	<0.001	0.180	<0.001
BMI *z*-score	0.168	<0.001	0.220	<0.001	0.120	<0.001
SUA, mg/dL	0.232	<0.001	0.240	<0.001	0.227	<0.001
FPG, mmol/L	0.014	0.446	0.018	0.432	0.024	0.430
Serum Cr, μmol/L	0.076	<0.001	0.093	<0.001	0.056	0.065
BUN, μmol/L	−0.029	0.116	−0.029	0.204	0.004	0.891
TC, mmol/L	−0.027	0.131	0.011	0.623	−0.097	0.001
LDL-C, mmol/L	0.152	<0.001	0.186	<0.001	0.087	0.004
AST, U/L	−0.176	<0.001	−0.185	<0.001	−0.154	<0.001
ALT, U/L	0.171	<0.001	0.191	<0.001	0.156	<0.001
ALP, U/L	0.138	<0.001	0.150	<0.001	0.112	<0.001
GGT, U/L	0.277	<0.001	0.329	<0.001	0.217	<0.001
White blood cells (10^3^/μL)	0.164	<0.001	0.181	<0.001	0.146	<0.001
Neutrophils (10^3^/μL)	0.161	<0.001	0.162	<0.001	0.173	<0.001
Monocyte (10^3^/μL)	0.109	<0.001	0.110	<0.001	0.137	<0.001
Lymphocyte (10^3^/μL)	0.072	<0.001	0.091	<0.001	0.031	0.300
Platelet (10^3^/μL)	0.104	<0.001	0.121	<0.001	0.080	0.008
NLR	0.085	<0.001	0.073	0.001	0.122	<0.001
dNLR	0.061	0.001	0.070	0.002	0.039	0.198
MLR	0.038	0.037	0.029	0.198	0.085	0.005
NMLR	0.082	<0.001	0.070	0.002	0.122	<0.001
SIRI (10^3^/μL)	0.118	<0.001	0.113	<0.001	0.156	<0.001
SII (10^3^/μL)	0.118	<0.001	0.113	<0.001	0.145	<0.001

In addition, hematological parameters (including WBC, neutrophil count, monocyte count, and platelet count), as well as CBC-derived inflammatory indices, such as NLR, dNLR, MLR, SIRI, and SII, showed a significantly positive correlation with the TG/HDL-C ratio (all *p* < 0.050).

### 3.3 The relationship between SUA and the TG/HDL-C ratio

**Figure 2:**
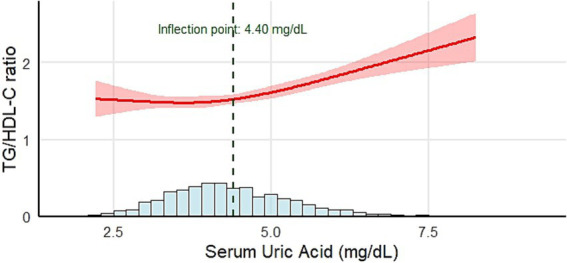
Relationship between SUA and the TG/HDL-C ratio. Adjustment variables: age, BMI *z*-score, AST, ALT, GGT, and ALP. BMI, body mass index; AST, aspartate aminotransferase; ALT, alanine aminotransferase; ALP, alkaline phosphatase; GGT, gamma-glutamyl transferase.

A restricted cubic spline (RCS) regression model was applied to conduct a piecewise analysis of the relationship between SUA and the TG/HDL-C ratio (Figure 2). The analysis revealed a nonlinear positive association between SUA and the TG/HDL-C ratio (*p* < 0.001, *p* for nonlinear = 0.008). When SUA < 4.40 mg/dL, a modest positive correlation with the TG/HDL-C ratio was observed (*β*: 0.14, 95% CI: 0.04 ~ 0.25, *p* = 0.006). When SUA ≥ 4.40 mg/dL, the correlation became significantly stronger (*β*: 0.40, 95% CI: 0.32 ~ 0.47, *p* < 0.001). When stratified by the SUA inflection point (4.4 mg/dL), the proportion of participants with a BMI *z*-score >2 and the prevalence of dyslipidemia were markedly higher in the higher SUA group than in the lower SUA group (Table 3). Collectively, these findings imply that elevated SUA beyond the 4.40 mg/dL threshold may serve as an early marker for increased susceptibility to overweight and dyslipidemia in children with adenoid or tonsillar hypertrophy.

**Table 3:** Comparison of anthropometric and lipid characteristics between children with SUA < 4.40 mg/dL and ≥4.40 mg/dL.

	SUA<4.40 mg/dL	SUA ≥ 4.40 mg/dL	*p*-value
*N*	1,641	1,385	–
Number of children (BMI *z*-score ≥2.0)	37 (2.2)	75 (5.4)	<0.001
TC, mmol/L	3.82 ± 0.65	3.83 ± 0.64	0.292
TG, mmol/L	0.84 ± 0.45	1.01 ± 0.60	<0.001
HDL-C, mmol/L	1.46 ± 0.31	1.36 ± 0.29	<0.001
LDL-C, mmol/L	2.09 ± 0.55	2.15 ± 0.55	<0.001
Hypercholesterolemia [*n* (%)]	48 (2.9)	35 (2.5)	0.504
Hypertriglyceridemia [*n* (%)]	108 (6.6)	201 (14.5)	<0.001
Low HDL-C [*n* (%)]	109 (6.6)	177 (12.8)	<0.001
High LDL-C [*n* (%)]	8 (0.5)	8 (0.6)	0.804
Dyslipidemia [*n* (%)]	221 (13.5)	318 (23.0)	<0.001

To further evaluate the robustness of the SUA inflection point, we applied data-driven two-piecewise linear regression with non-parametric bootstrap resampling (500 iterations). The bootstrap analysis yielded a mean inflection point of 4.61 mg/dL, with a 95% CI of 3.28–5.79 mg/dL, supporting the stability of the nonlinear transition region identified in the RCS model. Importantly, the bootstrap-derived confidence intervals overlapped with the inflection regions identified by the RCS models, supporting the robustness and consistency of the observed nonlinear relationship between SUA and the TG/HDL-C ratio.

### 3.4 The relationship between SUA and the TG/HDL-C ratio in different genders

**Figure 3:**
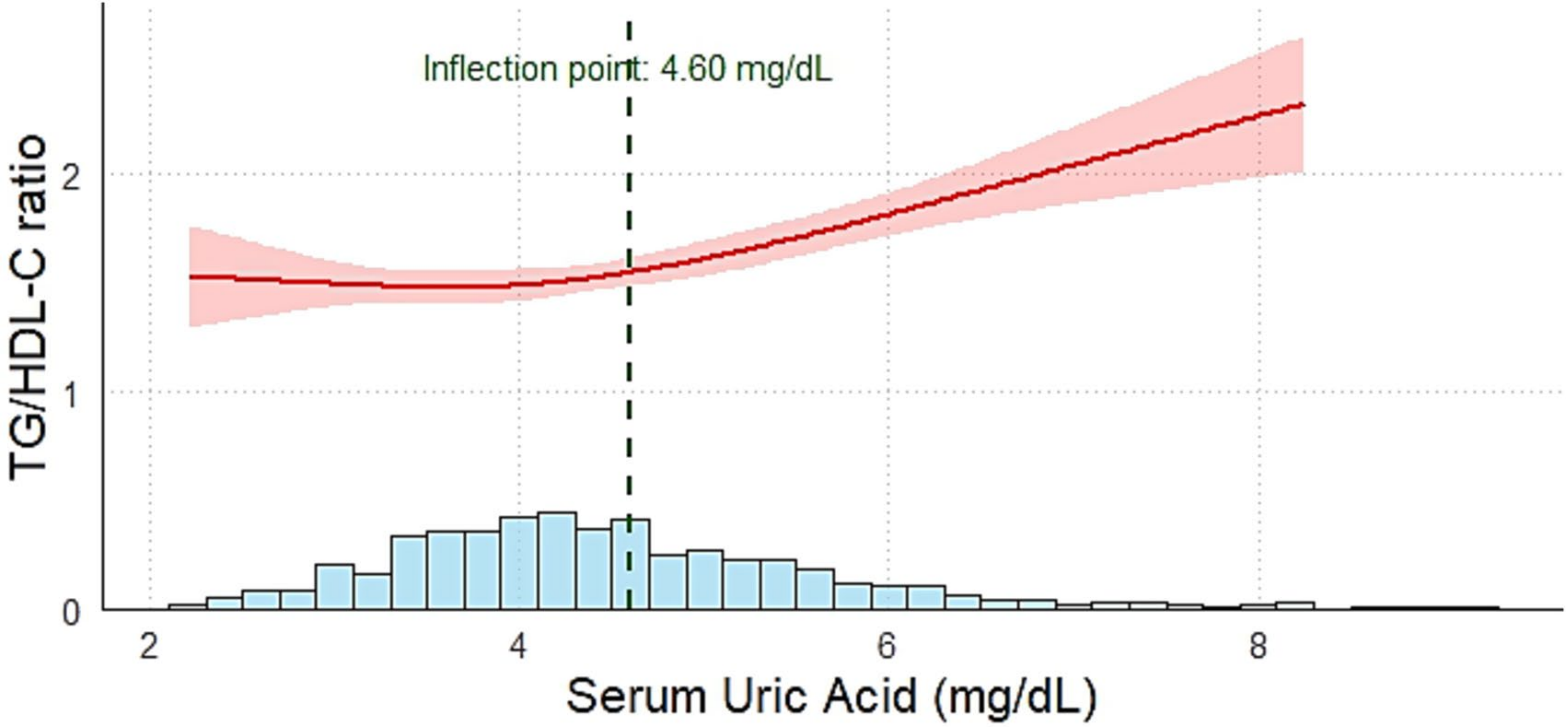
Relationship between SUA and the TG/HDL-C ratio in males. Adjustment variables: age, BMI *z*-score, AST, ALT, GGT, and ALP. BMI, body mass index; AST, aspartate aminotransferase; ALT, alanine aminotransferase; ALP, alkaline phosphatase; GGT, gamma-glutamyl transferase.

**Figure 4:**
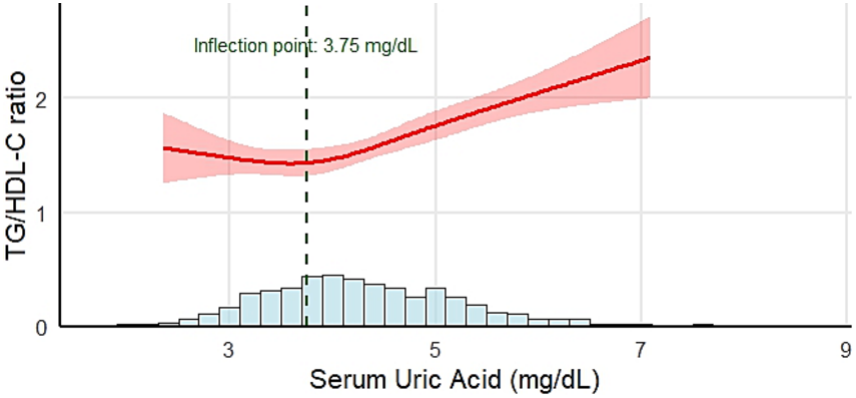
Relationship between SUA and the TG/HDL-C ratio in females. Adjustment variables: age, BMI *z*-score, AST, ALT, GGT, and ALP. BMI, body mass index, AST, aspartate aminotransferase; ALT, alanine aminotransferase; ALP, alkaline phosphatase; GGT, gamma-glutamyl transferase.

The RCS linear regression model was employed to conduct a piecewise regression of the association between SUA and the TG/HDL-C ratio across different sexes. The relationship between SUA and the TG/HDL-C ratio in boys and girls aligns with the pattern observed in the general population (boys: *p* = 0.002, *p* for nonlinear = 0.018; girls: *p*<0.001, *p* for nonlinear = 0.029). For boys, when SUA < 4.60 mg/dL, the TG/HDL-C ratio exhibited a modest positive correlation (*β*: 0.15, 95% CI: 0.03 ~ 0.27, *p* = 0.012). When SUA ≥ 4.60 mg/dL, the correlation became significantly stronger (*β*: 0.39, 95% CI: 0.29 ~ 0.49, *p* < 0.001) (Figure 3). For girls, when SUA < 3.75 mg/dL, there was no statistically significant correlation with the TG/HDL-C ratio (*β*: −0.07, 95% CI: −0.33 ~ 0.25, *p* = 0.18). In contrast, when SUA ≥ 3.75 mg/dL, a statistically significant positive correlation with the TG/HDL-C ratio was identified (*β*: 0.44, 95% CI: 0.34 ~ 0.53, *p* < 0.001) (Figure 4).

To further assess the robustness of the sex-specific inflection points observed in the RCS analyses, we conducted bootstrap-based two-piecewise linear regression analyses (500 iterations) for boys and girls separately. Among boys, the bootstrap procedure yielded a mean inflection point of 4.61 mg/dL (95% CI: 3.28–5.79 mg/dL). Among girls, the corresponding mean inflection point was 3.94 mg/dL (95% CI: 3.27–5.35 mg/dL).

## 4 Discussion

In the present study, we evaluated the association between SUA levels and the TG/HDL-C ratio in school-age children with adenoid or tonsillar hypertrophy. Notably, a nonlinear relationship was observed, with the SUA inflection point identified at 4.40 mg/dL. Above this level, the positive relationship between SUA and the TG/HDL-C ratio was stronger compared to that below the inflection point. Furthermore, when SUA levels exceeded 4.40 mg/dL, the proportion of children with a BMI *z*-score greater than 2.0 and the prevalence of dyslipidemia were significantly higher than those in the lower SUA groups. Additionally, sex-stratified analysis revealed that the relationship between SUA and the TG/HDL-C ratio in both boys and girls was consistent with that in the general population. However, the inflection points differed significantly between sexes, with inflection points of approximately 4.60 mg/dL in boys and 3.75 mg/dL in girls.

In our study, participants in higher SUA quartiles exhibited significant increases in BMI *z*-scores, TG, TC, and LDL-C levels, while HDL-C levels decreased progressively. These findings align with those of a 9-year follow-up study of nearly 16,000 Chinese adults, which demonstrated that elevated SUA levels were significantly associated with a higher risk of obesity, particularly among women and younger individuals (13). Similarly, in children aged 3–12 years with adenoid or tonsillar hypertrophy, SUA was independently associated with dyslipidemia, and an SUA level below 1 SD of the mean value observed in participants with normal lipid profiles was associated with a healthier lipid profile (14). This highlights the potential role of SUA as an early marker of lipid-related metabolic health across different age groups.

The TG/HDL-C ratio has been widely used to assess insulin resistance, lipid imbalance, and cardiovascular risk (7). In China, a cross-sectional study of 262 obese children in Beijing demonstrated that the TG/HDL-C ratio is a useful marker for predicting insulin resistance among children aged 6–9 years and 10–13.5 years (5). Moreover, a study conducted among children in Argentina also reported that the TG/HDL-C ratio is a reliable indicator for identifying insulin resistance in pediatric populations (15). In hyperuricemia patients, Li et al. (16) observed an association between SUA and the TG/HDL-C ratio, suggesting its predictive value as an indicator of metabolic disorders. Elevated SUA levels are closely associated with the disruption of lipid metabolism, characterized by increased TG and decreased HDL-C. SUA may influence lipid imbalance through several mechanisms: (1) SUA promotes oxidative stress and inflammation, which can exacerbate triglyceride synthesis in the liver and reduce HDL-C levels (17); (2) hyperuricemia has been linked to adiponectin suppression, a hormone crucial for lipid oxidation and HDL-C maintenance, and low adiponectin levels contribute to increased TG levels and reduced HDL-C, elevating the TG/HDL-C ratio (18); (3) SUA-induced oxidative stress impairs endothelial function by reducing nitric oxide bioavailability, disrupting lipid metabolism, and promoting atherogenic lipid profiles (19). The strong positive correlations of SUA with the TG/HDL-C ratio reinforce its role as a robust indicator of insulin resistance and lipid dysregulation. Although our findings support the potential application of these indices in the pediatric population at risk for metabolic disorders, the underlying mechanisms could not be directly validated using available data.

One of the most notable findings of this study was the nonlinear relationship between SUA and the TG/HDL-C ratio observed using the RCS model. When SUA ≥ 4.40 mg/dL, the positive association with the TG/HDL-C ratio became stronger compared with levels below this inflection point. These results are consistent with the findings of Han et al., who observed similar nonlinear trends in hyperuricemia patients (20). In our study, the inflection point in boys was slightly higher than that observed in the entire population. For girls, the inflection point was 3.75 mg/dL, which was significantly lower than that for boys. This result reveals that girls are more sensitive to SUA levels than boys. This also indicates that stricter standards should be applied when managing SUA levels in girls, as even a slight increase in lower uric acid levels can impact metabolic health.

Furthermore, children with SUA levels >4.40 mg/dL exhibited a significantly higher proportion of BMI *z*-scores > 2.0 and a greater prevalence of dyslipidemia than those with lower SUA levels. This finding suggests that elevated SUA levels may be closely linked to adverse metabolic phenotypes, including obesity and lipid dysregulation, even in school-aged children. Similarly, a national survey showed that SUA was positively associated with all lipid components and that the number of dyslipidemia components increased with increasing SUA levels (21). In animal models, hyperuricemia disrupts lipid metabolism through upregulation of the CXCL-13 pathway (22). Clinically, this data-driven inflection point of SUA (≥4.40 mg/dL) may serve as a simple risk flag during routine laboratory screening for children with adenoid or tonsillar hypertrophy. When elevated SUA levels are detected, further evaluation of metabolic parameters such as insulin resistance, body composition, and lifestyle factors (diet, physical activity, and sleep patterns) should be considered. Given the observed sex-specific patterns in SUA inflection points, future large-scale and longitudinal studies are required to establish sex-specific reference thresholds and externally validate these findings.

A major strength of this study is its large sample size and focus on the pediatric population, offering valuable insights into an area with limited research. Additionally, the application of the RCS model for piecewise analysis allows for a more detailed exploration of the nonlinear relationships between SUA levels and the TG/HDL-C ratio. However, this study has several limitations. First, the retrospective design prevents causal inferences, and the reliance on a single population restricts the generalizability of the findings. Second, our study lacked data on potential confounders, including dietary patterns, physical activity levels, and objective indicators of tonsillar or adenoidal hypertrophy, indicating that some potential confounding factors could not be completely ruled out. Third, SUA and lipid parameters were measured only once, which may not fully capture intra-individual variability or long-term metabolic status. Future prospective, longitudinal, and multicenter studies are necessary to validate the SUA inflection point identified in this study, confirm the reproducibility of our findings, and further elucidate the underlying causal pathways in diverse pediatric populations. In addition, mechanistic and interventional studies are needed to determine whether reducing SUA levels can improve lipid profiles and overall metabolic health of children.

## 5 Conclusion

Our study identified a nonlinear relationship between SUA and the TG/HDL-C ratio in school-age children with adenoid or tonsillar hypertrophy. This nonlinear pattern persisted in the sex-stratified analysis, with a higher inflection point in boys. These findings suggest that SUA could serve as a practical marker for early metabolic risk assessment in this pediatric population.

## Data Availability

The raw data supporting the conclusions of this article will be made available by the authors, without undue reservation.

## Footnotes

1
https://www.R-project.org
